# A Marine Diterpenoid Modulates the Proteasome Activity in Murine Macrophages Stimulated with LPS

**DOI:** 10.3390/biom8040109

**Published:** 2018-10-05

**Authors:** Yisett González, Deborah Doens, Héctor Cruz, Ricardo Santamaría, Marcelino Gutiérrez, Alejandro Llanes, Patricia L. Fernández

**Affiliations:** 1 Centro de Biología Celular y Molecular de Enfermedades, Instituto de Investigaciones Científicas y Servicios de Alta Tecnología (INDICASAT AIP), Edificio 219, Ciudad del Saber, 0801 Panamá, Panama; 2 Facultad de Ciencias de la Salud Dr. William C. Gorgas, Universidad Latina de Panamá, 0801 Panamá, Panama; 3 Centro de Biodiversidad y Descubrimiento de Drogas, INDICASAT AIP, Edificio 219, Ciudad del Saber, 0801 Panamá, Panama

**Keywords:** marine diterpenoid, proteasome inhibitors, immunoproteasome

## Abstract

The proteasome is an intracellular complex that degrades damaged or unfolded proteins and participates in the regulation of several processes. The immunoproteasome is a specialized form that is expressed in response to proinflammatory signals and is particularly abundant in immune cells. In a previous work, we found an anti-inflammatory effect in a diterpenoid extracted from the octocoral *Pseudopterogorgia acerosa*, here called compound 1. This compound prevented the degradation of inhibitor κB α (IκBα) and the subsequent activation of nuclear factor κB (NFκB), suggesting that this effect might be due to inhibition of the ubiquitin-proteasome system. Here we show that compound 1 inhibits the proteasomal chymotrypsin-like activity (CTL) of murine macrophages in the presence of lipopolysaccharide (LPS) but not in its absence. This effect might be due to the capacity of this compound to inhibit the activity of purified immunoproteasome. The compound inhibits the cell surface expression of major histocompatibility complex (MHC)-I molecules and the production of proinflammatory cytokines induced by LPS in vitro and in vivo, respectively. Molecular docking simulations predicted that compound 1 selectively binds to the catalytic site of immunoproteasome subunits β1i and β5i, which are responsible for the CTL activity. Taken together these findings suggest that the compound could be a selective inhibitor of the immunoproteasome, and hence could pave the way for its future evaluation as a candidate for the treatment of inflammatory disorders and autoimmune diseases.

## 1. Introduction

The proteasome is an enzymatic complex found in the nucleus and cytoplasm of eukaryotic cells, archaea and certain bacteria. This complex is responsible for the degradation of intracellular proteins that are damaged or misfolded. It works in collaboration with the ubiquitin system, which tags proteins for proteasome processing. The proteasome plays an important role in the regulation of many cellular processes, such as the cell cycle, the defense against oxidative stress and inflammatory responses. The proteasome is composed of two types of domains: a core particle and one or two regulatory domains. The core particle is formed by four stacking rings, each of them consisting of seven α or β subunits. Central rings have three catalytic subunits, namely β1, β2 and β5, which have caspase-like, trypsin-like and chymotrypsin-like (CTL) activity, respectively. An alternative form of the proteasome, called immunoproteasome, is present in most animal cells but it is abundantly expressed in immune cells, where its primary role is to process proteins for antigen presentation by major histocompatibility complex (MHC) class I molecules [1,2]. Expression of the immunoproteasome is induced by interferon-γ (IFN-γ), tumor necrosis factor (TNF) and bacterial lipopolysaccharide (LPS) under inflammatory conditions, such as infections or autoimmune diseases [3,4,5]. In the presence of such stimuli, catalytic subunits of the constitutive form are respectively substituted by inducible subunits β1i (LPM2), β2i (MECL-1) and β5i (LMP7) to form the immunoproteasome. Unlike its constitutive counterparts, which have caspase-like activity, the β1i subunit also has CTL activity [6,7].

The proteasome has been implicated as a modulator of inflammatory responses by participating in the activation of nuclear factor κB (NFκB), a transcription factor that regulates the expression of many genes involved in inflammation [8]. Five NFκB family members have been described, namely RelA (p65), RelB, cRel, p50 and p52, respectively encoded by genes *rela, relb, crel, nfkb1* and *nfkb2*. After the stimulus, NFκB proteins form dimers, which bind to κB sites on target genes either as homodimers or heterodimers. In resting cells, NFκB is sequestered in the cytoplasm by inhibitor κB (IκB) proteins. Activation of NFκB is triggered by phosphorylation of IκB, followed by its ubiquitination and proteasomal degradation, thus releasing NFκB and promoting its translocation into the nucleus [9]. It has been demonstrated that the immunoproteasome subunit β1i is involved in the proteolytic processing of NFκB precursor proteins (p100/p105), as well as in the degradation of inhibitor κB α (IκBα) [10,11,12]. Later, it was observed that β1i-deficient retinal pigment epithelial cells exhibited diminished activation of NFκB in response to TNF [13]. However, the role of the immunoproteasome in NFκB activation and in the degradation of IκB proteins is still under debate [10,14,15,16,17]. Other studies have demonstrated that immunoproteasome subunits are not essential in the activation of NFκB either in cancer cell lines or in peritoneal macrophages stimulated with TNF [17,18].

Due to the role of the proteasome in many physiological processes, it has become a major target for the design of new drugs as a therapeutic for several diseases. Many proteasome inhibitors have been identified from natural and synthetic sources. Two of them, bortezomib and carfilzomib, are currently approved for the treatment of multiple myeloma. Although a number of second-generation proteasome inhibitors are in clinical trials [19], undesirable side effects have been associated to these molecules. The immunoproteasome has emerged as a therapeutic target and as a strategy to reduce the toxicity associated with the inhibition of the constitutive proteasome in cells [20,21]. These molecules are not only valuable as potential therapeutics but would also allow a better understanding of the physiological roles attributed to the immunoproteasome. Several highly selective immunoproteasome inhibitors have been recently described, including both peptidic [22] and nonpeptidic inhibitors [23].

In previous studies, we have shown a marked anti-inflammatory activity for a pseudopterane diterpene (compound 1) isolated from the octocoral *Pseudopterogorgia acerosa* [16]. Compound 1 inhibited the production and expression of proinflammatory mediators in macrophages stimulated with LPS, TNF and other toll-like receptor ligands. Our results showed that this anti-inflammatory effect is due to the inhibition of IκBα degradation and the subsequent activation of NFκB. We then analyzed if the effect of compound 1 might be influenced by a modulation of the ubiquitin-proteasome system, affecting the proteasomal degradation of phosphorylated IκBα. We show herein that compound 1 inhibits the CTL activity of the proteasome induced by LPS in vitro and reduces the expression of MHC class I in macrophages. This inhibitory effect might occur by a mechanism that involved the modulation of immunoproteasome activity, since a reduction in the CTL activity of the purified immunoproteasome was observed. In vivo, compound 1 reduces the production of proinflammatory mediators in the lung of animals treated by intranasal inoculation of LPS. Molecular docking simulations predicted that compound 1 preferentially interacts with the catalytic site of subunits β1i and β5i, suggesting that the effect of this compound might be dependent on immunoproteasome activity.

## 2. Materials and Methods

### 2.1. Mice

In vivo studies were carried out by using female C57Bl/6 mice with an age of eight weeks, obtained from Instituto de Investigaciones Científicas y Servicios de Alta Tecnología (INDICASAT)’s mouse facility. Mice were kept at 25 °C under a light/dark cycle of 12 h and had free access to food and water. All experiments were performed in accordance with guidelines from the Institutional Animal Welfare Committee and the Guide for the Care and Use of Laboratory Animals of the National Institutes of Health. The protocol was also approved by the Institutional Animal Care and Use Committee of INDICASAT AIP (IACUC-15-004).

### 2.2. Acute Pulmonary Inflammation

C57BL/6 mice (*n* = 5) were anesthetized with Ketamine/Xylazine (93/6 mg/Kg) and then treated by intranasal inoculation with lipopolysaccharide (LPS) from *Escherichia coli* 0111:B4 (Sigma Aldrich, Saint Louis, MO, USA) (0.5 mg/Kg) or saline for control group. Compound 1 (5 mg/Kg) was administered by intraperitoneal (i.p.) injection 2 h before and 10 h after LPS administration. The control group was not treated with compound 1. Mice were euthanized 24 h after the challenge with LPS and the concentrations of tumor necrosis factor (TNF) and interleukin (IL)-6 were determined in lungs and in bronchoalveolar lavage (BAL) by the enzyme-linked immunosorbent assay (ELISA) method. The expression of proteasome and immunoproteasome subunits was determined by quantitative polymerase chain reaction (PCR) in whole lung homogenate of animals from LPS and control groups.

### 2.3. Cell Culture and Proteasome Activity Assay

Peritoneal macrophages from C57BL/6 mice were obtained five days after intraperitoneal instillation of 2 mL of thioglycollate 3%, by peritoneal washing with chilled Roswell Park Memorial Institute (RPMI) medium. Cells were seeded in RPMI with 10% fetal calf serum (FCS) at a density of 1 × 10^6^/well in 24-well plates and cultured for 2 h at 37 °C in an atmosphere of 5% CO_2_. Non-adherent cells were removed by washing and adherent cells were pre-incubated with compound 1 (25 μM) or isogorgiacerodiol (25 or 50 μM) for 2 h at 37 °C in an atmosphere of 5% CO_2_. Then, cells were stimulated with bacterial LPS from *Escherichia coli* 0111:B4 (InvivoGen, San Diego, CA, USA) (1 μg/mL) for different periods of time (2, 4 or 8 h). Supernatants were discarded and cells were incubated with the fluorogenic peptide Suc-Leu-Leu-Val-Tyr-AMC to evaluate the proteasome CTL activity or Z-Leu-Leu-Glu-AMC to evaluate caspase-like activity as previously described [24,25]. After 2 h, supernatants were harvested and the fluorescence of free fluorophore 7-amino-4-methycoumarin (AMC) was measured by using FLx800 BioTek (Winooski, VT, USA) at wavelength/band pass 360/40 for excitation and 460/40 for emission.

### 2.4. Western Blot Analysis

Western blot analysis was performed as previously described by González et al. [16]. Briefly, peritoneal macrophages were stimulated with 1 μg/mL of LPS with or without 25 μM of compound 1. Cells were further lysed and 20 μg of total extracts were diluted in loading buffer, boiled and applied to a sodium dodecyl sulfate (SDS) polyacrilamide gel (12%) under reducing conditions. Protein bands were transferred to a polyvinylidene difluoride (PVDF) membrane and further incubated overnight with a monoclonal antiubiquitin antibody specific for Lys48 [26]. For Western blot image densitometry, ImageJ v. 1.50i [27] was used as recommended by the software developer.

### 2.5. Major Histocompatibility Complex Class I Flow Cytometry Analysis

The experiments of cell surface quantification of MHC-I expression were performed in bone marrow-derived macrophages (BMDM), since these cells have lower levels of basal MHC-I expression than elicited peritoneal macrophages. The BMDM were extracted and cultured as previously described by González et al. [16]. Cultured cells were stimulated with LPS at a concentration of 1 μg/mL in the presence or absence of compound 1 (25 μM). After 24 h, expression of MHC-I was evaluated by flow cytometry.

After 24 h of stimulus, cells were collected, washed with phosphate-buffered saline (PBS) and blocked for 15 min with 200 µL bovine serum albumin (BSA) 1% in PBS. Cells were then washed and incubated for 30 min at 4 °C with antimouse CD11b fluorescein isothiocyanate (FITC) (5 µg/mL) and/or phycoerythrin (PE) antimouse H-2Ld/H-2Db clone 28-14-8 (Biolegend, San Diego, CA, USA) diluted in PBS BSA 1%. After several washes, cells were resuspended in PBS and analyzed by flow cytometry. Event acquisition was performed with a Partec CyFlow^®^ cytometer and the data were analyzed using FlowMax software (PARTEC, Münster, Germany) and FCS Express 4 Flow Cytometry (De Novo software, Los Angeles, CA, USA).

### 2.6. Quantitative Real Time Polymerase Chain Reaction

Elicited peritoneal macrophages were stimulated with LPS (10 ng/mL) for 2, 4 and 8 h or preincubated for 1 h with compound 1 (25 μM), PR-957 (200 nM) or Polymyxin B (15 μg/mL) and then stimulated with LPS (10 ng/mL) for 8 h. After the stimuli, total RNA was extracted using TRIzol (Life Technology Corporation: Invitrogen and Applied Biosystems, Foster City, CA, USA). The cDNA was obtained by using a High-Capacity cDNA Reverse Transcription Kit (Life Technology Corporation: Invitrogen and Applied Biosystems). The ABI 7500 (Applied Biosystems) was used to perform the quantitative real-time PCR analysis using SYBR Green master mix (Applied Biosystems). Amplification conditions were: 95 °C (10 min), 40 cycles of 95 °C (15 s), and 59 °C (60 s). The data were normalized to the hypoxanthine phosphoribosyltransferase (HPRT) expression and were represented as the difference relative to the control level. The 2^-∆∆^CT method was used to analyze the relative gene expression. The following forward/reverse primer pairs were used: 5’-TGACCAAGGACGAATGTCTG-3’/5’-GATTTGGTCTCCCAAAAGCA-3’ for β1; 5’-GTGAATCAGCACGGGTTTT-3’/5’-AATCCGCTGCAACAATGACT-3’ for β5; 5’-CATCATGGCAGTGGAGTTTGAC-3’/5’-ACCTGAGAGGGCACAGAAGATG-3’ for β1i; 5’-ACCACACTCGCCTTCAAGTTC-3’/5’-GCCAAGCAGGTAAGGGTTAATC-3’ for β5i and 5’-GCTGGTGAAAAGGACCTCT-3’/5’-CACAGGACTAGAACACCTGC-3’ for HPRT.

### 2.7. Purified Proteasome Activity Assay

The assay was performed by using the Proteasome-Glo^TM^ Assay Systems (Promega, Madiscon, WI, USA) following the manufacturer’s instructions. Briefly, 2 nM of mouse 20S proteasome or immunoproteasome (Boston Biochem, Cambridge, MA, USA) were added to a white 96-well plate in a volume of 50 μL. Then, 50 μL of compound 1 was added at different concentrations (50, 25 and 12.5 μM) and the plate was incubated for 1 h at 37 °C. Following, 50 μL of the Proteasome-Glo^TM^ reagent containing the luciferin detection reagent and the substrates (Suc-LLVY for Chymotrypsin-like or Z-nLPnLD for Caspase-like) were added. The plate was mixed at 300–500 rpm for 30 s and incubated at room temperature for 30 min. Luminescence was measured by using a plate reader Synergy HT from BioTek.

### 2.8. Cytokine Measurements

Animals were euthanized and the BAL was obtained by injecting 1 mL of PBS into the trachea and collecting again. The BAL was centrifuged and the supernatants were harvested. The whole lung homogenate was obtained by homogenization of the tissue in 1X PBS containing 0.1% of Triton100 and protease inhibitors. Homogenates were centrifuged and the supernatants were stored. The concentrations of TNF and IL-6 in lungs and BAL were determined by ELISA (DuoSet kit, R&D System, Minneapolis, MN, USA), according to the manufacturer’s protocol.

### 2.9. Molecular Docking Simulation

ACD/ChemSketch v. 12 (ACD/Labs, Toronto, Canada) was used to draw the 2D structures of compound 1 and isogorgiacerodiol, and to convert them into 3D structural data. Avogadro v.1.1.1 [28] was used to optimize the geometry of both molecules. Antechamber v.1.27 [29] was used for an additional energy minimization step before docking. For preparation of the receptor protein, we isolated the dimer formed by chains K and L from the murine constitutive proteasome (Protein Data Bank (PDB) accession code 3UNB) and immunoproteasome (PDB accession code 3UNF), corresponding to subunits β5/β5i and β6, respectively. All ligands and water molecules were removed before docking. The co-crystallized ligand in both structures was used to define the position of the binding site of subunits β5/β5i and to set grid parameters for docking. Docking simulations per se were performed with Dock v. 6.7 [30] by using grid score and Hawkings GB/SA as primary and secondary energy scoring functions, respectively. The program was set to output the best 10 docking poses and those with the lowest energy were selected for further analyses in each experiment. Prediction of hydrogen bonds and other noncovalent interactions were done with Chimera v.1.11 [31] and LigPlot+ [32].

### 2.10. Statistical Analysis

Data are presented as means + standard error of the mean (SEM) or mean + standard deviation (SD). Results were analyzed using a statistical software package (GraphPad Prism 6). Statistical analyses were performed by unpaired *t* test, Mann-Whitney test, Kruskal-Wallis multiple comparisons test. A difference between groups was considered to be significant if *p* < 0.05 (* *p* < 0.05; ** *p* < 0.01; *** *p* < 0.001). The half maximal inhibitory concentration (IC_50_) was calculated adjusting a sigmoidal dose−response curve following GraphPad Prism 6 procedure.

## 3. Results

### 3.1. Compound 1 Inhibits the Activity of the Proteasome with the Consequent Accumulation of Ubiquitinated Proteins

**Figure 1:**
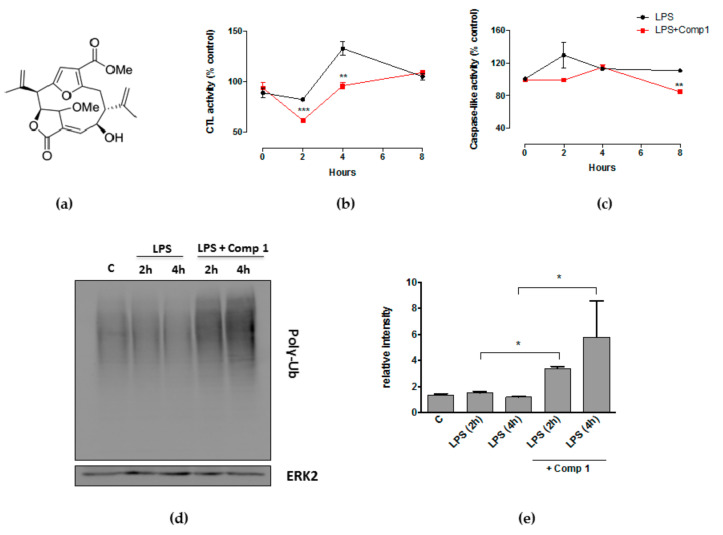
Compound 1 inhibits proteasome activity in the presence of lipopolysaccharide (LPS). (**a**) Schematic representation of compound 1. Macrophages were stimulated with LPS (1 μg/mL) in the presence or absence of compound 1 (25 μM) for 2, 4 or 8 h. Hydrolysis of fluorogenic peptides Suc-Leu-Leu-Val-Tyr-AMC (**b**) or Z-Leu-Leu-Glu-AMC (**c**) was measured in cell supernatants by detection of free 7-amino-4-methycoumarin (AMC). Results were normalized with DMSO-treated controls. Results represent mean ± standard error of the mean (SEM) from treatments performed in triplicates and are representative of two different experiments. (**d**) Immunoblotting for poly-Ub detection in macrophage extracts obtained from cells stimulated with LPS in the presence or absence of compound 1. The Anti-Ubiquitin Lys48 antibody was used to detect poly-Ub. Detection of ERK2 was used as a loading control. The figure is representative of two different experiments with similar results. (**e**) Relative intensity calculated by the ratio of area under the curve from poly-Ub/area under the curve for total. Results represent mean + SEM from two different experiments * *p* < 0.05; ** *p* < 0.01; *** *p* < 0.001 compared to LPS alone. CTL: chemotrypsin-like.

We have previously demonstrated that compound 1 (Figure 1a) inhibits the degradation of IκBα, leading to the prevention of NFκB activation and the subsequent transcription of genes encoding pro-inflammatory mediators [16]. Considering the effect of compound 1 on the degradation of IκB, we determined if it interferes with the ubiquitin-proteasome system. We first analyzed if compound 1 modulates the proteasome CTL and caspase-like activities, which have been largely implicated in the regulation of immune responses [33]. Macrophages were treated with LPS and compound 1 for different time points and then incubated with the fluorogenic peptides Suc-Leu-Leu-Val-Tyr-AMC or Z-Leu-Leu-Glu-AMC, substrates for CTL and caspase-like activities respectively. Treatment with LPS induced an increase in CTL activity after 4 h of stimulus that was maintained until 8 h. Compound 1 considerably reduced CTL activity 2 and 4 h after treatment (Figure 1b). Compound 1 also inhibited the caspase-like activity at 8 h of stimulus (Figure 1c). Although the compound has no apparent effect on the CTL activity in the absence of stimulus with LPS, it significantly inhibits caspase-like activity (Figure S5). The inhibitory effect of compound 1 is not due to cell death since we have previously demonstrated that compound 1 is not cytotoxic [16]. The effect of compound 1 on proteasome activity was also evidenced by the accumulation of polyubiquitinated (poly-Ub) proteins in macrophages stimulated with LPS (Figure 1d). The accumulation of poly-Ub proteins was detected by immunoblotting in macrophage extracts obtained from cells stimulated with LPS in the presence or absence of compound 1. Western blot analysis shows a notably higher accumulation of poly-Ub proteins in the presence of compound 1 than in its absence (Figure 1d,e). These results suggest that compound 1 might have an effect on proteasome activity.

### 3.2. Compound 1 did not Affect the Expression of β1i and β5i Subunits in Peritoneal Macrophages Stimulated with Lypopolisaccharide

It has been previously demonstrated that LPS induces the expression of immunoproteasome subunits [5]. Then, we evaluated, in our experimental conditions, the capacity of LPS to induce the expression of the subunits involved in the CTL activity of the immunoproteasome, β1i and β5i, and their counterparts in the constitutive proteasome, β1 and β5. We stimulated peritoneal macrophages with LPS at different time points and the expression of the subunits was evaluated by quantitative PCR. The LPS treatment significantly increased the expression of β1i and β5i 4 and 8 h after the stimuli (Figure S6). We did not find statistically significant differences in the level of expression of both subunits induced by LPS (Figure S6). Although there is a slight increase in the expression of the constitutive subunits β1 and β5 induced by LPS, compared to nonstimulated controls, the levels of expression of these subunits were fivefold less than the expression of β1i and β5i (Figure S6).

**Figure 2:**
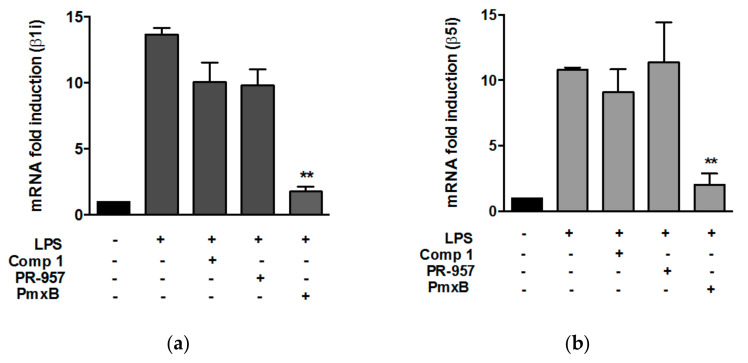
Compound 1 does not appear to interfere with the expression of immunoproteasome subunits. Peritoneal macrophages were pre-treated with compound 1 (25 μM), PR-957 (200 nM) or Polimixin B (PmxB) (15 μg/mL) and then stimulated with LPS (1 ng/mL) for 8 h. Total RNA was isolated and the amount of mRNA was determined for β1i (**a**) or β5i (**b**). Values were normalized by using hypoxanthine phosphoribosyltransferase (HPRT) expression and are shown as fold induction of mRNA expression with respect to control samples. Results represent means + SEM from two independent experiments performed in duplicates, ** *p* < 0.005.

We then analyzed if compound 1 interferes with the expression of proteasome subunits. Macrophages were treated with LPS for 8 h in the presence or absence of compound 1. The expression of the subunits induced by LPS was not affected by the treatment with compound 1 (Figure 2a, b). A known inhibitor of the immunoproteasome, PR-957, did not interfere with the expression of β1i and β5i either. As expected, the pretreatment of cells with polymyxin B, which binds to lipid A and interferes with the binding of LPS to TLR4, completely abrogated the increase of β1i and β5i expression induced by LPS (Figure 2a,b). The expression of β1 and β5 was not affected by the treatment of cells with compound 1 (data not shown).

### 3.3. Compound 1 Inhibits the Chemotrypsin-like Activity of Purified Proteasomes

**Figure 3:**
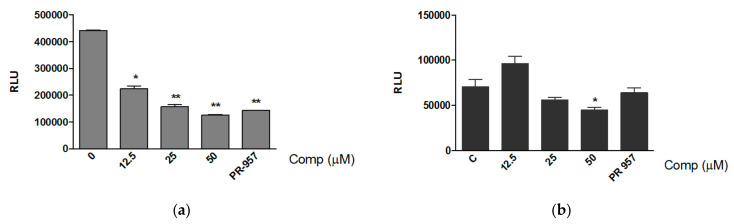
Compound 1 inhibits the activity of immunoproteasome at low concentration. Purified immunoproteasome (**a**) and constitutive proteasome (**b**) (2 nM) were incubated with different concentrations of compound 1 (12.5, 25 and 50 μM) or PR-957 (200 nM) at 37 °C. After 1 h the substrate Suc-LLVY was added and the luminescence was reported as relative light units (RLU). Results represent mean + standard deviation (SD) from treatments performed in triplicates.

In order to rule out the possibility of an unspecific effect of compound 1 on proteasome activity, we evaluated if the compound inhibits the activity of purified constitutive proteasome and immunoproteasome. Purified proteasomes were incubated with compound 1 at different concentrations and the CTL activity was determined after the addition of Suc-LLVY substrate. Compound 1 significantly reduced the activity of the immunoproteasome even at a concentration of 12.5 μM (Figure 3a), with an IC_50_ value of 9.767 μM (Figure S7). The compound also reduced the CTL activity of the constitutive proteasome at the concentration of 50 μM (Figure 3b). We did not observe any effect of compound 1 on the caspase-like activity of the constitutive proteasome (data not shown).

### 3.4. Compound 1 Inhibits the Expression of MHC-I In Vitro and the Production of Pro-Inflammatory Mediators In Vivo

**Figure 4:**
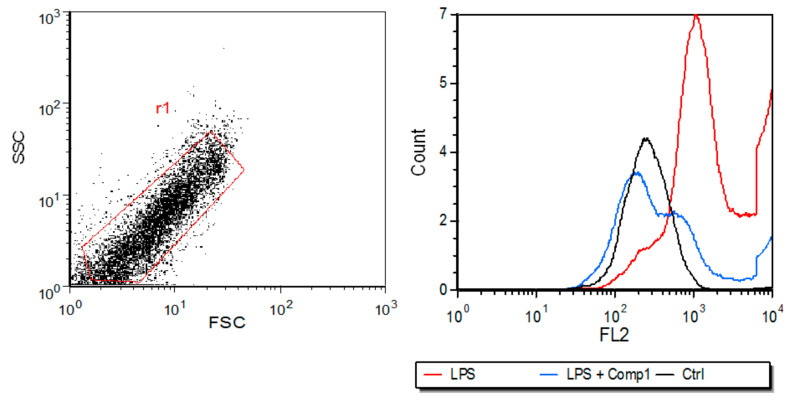
Expression of major histocompatibility complex (MHC)-I induced by LPS in macrophages appears to be inhibited by compound 1. LPS (1 μg/mL) was used to stimulate bone marrow-derived macrophages in the presence or absence of compound 1 (25 μM). Expression of MHC-I (H-2Ld/H-2Db) was measured on CD11b+ cells after 24 h. Figure shows a histogram of MHC-I expression representative of two different experiments. Control (Ctrl) corresponds to non-stimulated cells.

Immunoproteasome has been implicated in the presentation of certain classes of peptides by the MHC class I. The expression of MHC class I is also modulated by the immunoproteasome [2]. As we believe that compound 1 might be affecting immunoproteasome activity, we then analyzed if the compound interferes with the expression of MHC class I induced by LPS in macrophages. We stimulated bone marrow-derived macrophages with LPS and treated with compound 1. The levels of MHC-I on cell surface were analyzed by flow cytometry after 24 h of treatments. The histogram shows that LPS induced an increase in the levels of MHC-I expressed on macrophages surface, and this effect was reverted by the treatment of cells with compound 1 (Figure 4). The inhibition of MHC-I expression might be due to immunoproteasome modulation induced by compound 1.

**Figure 5:**
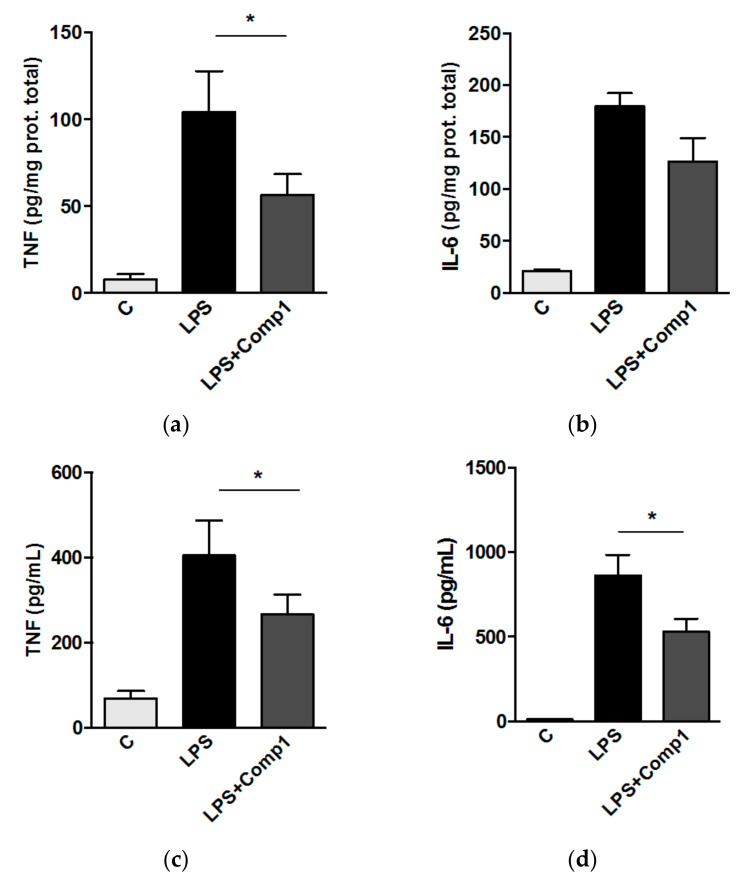
Compound 1 inhibits the production of pro-inflammatory mediators in vivo. C57BL/6 mice were injected intraperitoneal (i.p.) with compound 1 (5 mg/Kg), 2 h before and 10 h later after LPS (0.5 mg/Kg) inoculation. After 24 h of LPS treatment, protein levels of tumor necrosis factor (TNF) and interleukin (IL)-6 were quantified in lung tissue (**a**,**b**) and bronchoalveolar lavage (**c**,**d**). Results represent mean + SEM from two different experiments *, *p* < 0.05.

It has been observed that the selective inhibition of the immunoproteasome downregulates the expression of proinflammatory mediators in a variety of immune pathologies [34]. Hence, we determined whether compound 1 has an effect on the inflammatory response induced by LPS in vivo. Mice were treated with compound 1 by intraperitoneal (i.p.) administration 2 h before and 10 h later after intranasal inoculation of LPS. We first evaluated the expression of immunoproteasome subunits induced by LPS and we observed higher levels of β1i and β5i in the animals treated with LPS, compared with the control group (Figure S8). These levels were correlated with increased production of inflammatory mediators in lungs and BAL (Figure 5). Compound 1 significantly reduced the production of TNF in the lungs of animals treated with LPS (Figure 5a,b). Although the reduction of IL-6 in lungs was not statistically significant, we have observed a conserved trend in the experiments. The suppression of TNF and IL-6 in the presence of compound 1 was also observed in the bronchoalveolar lavage of treated animals (Figure 5c,d). LPS treatment did not increase the expression of β1 and β5 subunits from the constitutive proteasome (Figure S8). Thus, the effect of compound 1 in the inflammatory response in vivo could occur, at least in part, by means of the modulation of immunoproteasome activity.

### 3.5. Compound 1 Appears to Bind to the Catalytic Pocket of β5i Subunit of the Immunoproteasome

**Figure 6:**
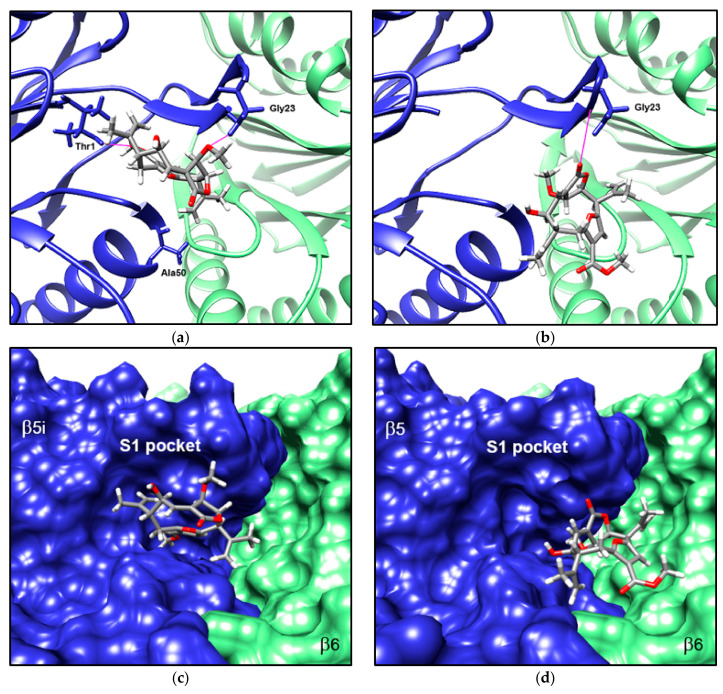
Molecular docking simulation predicted specific binding of compound 1 to the catalytic site of subunit β5i of the murine immunoproteasome. Lowest-energy pose predicted for the interaction of compound 1 with subunits β5i-β6 of the immunoproteasome (**a**) and with subunits β5-β6 of the constitutive form (**b**). Orientation of compound 1 within the S1 pocket of subunits β5i (**c**) and β5 (**d**) is also shown. Subunits β5/β5i are colored blue and β6 subunits are colored green. Purple lines indicate predicted hydrogen bonds between the ligand and amino acid residues from the subunits.

We used molecular docking simulation to further study the binding of compound 1 to the catalytic site of β5i subunit of the murine proteasomes. It has been previously shown that neighboring subunits contribute to interactions within the binding sites of proteasomal catalytic subunits [35]. Hence, we also included subunit β6 when performing the docking simulation for binding sites of subunits β5 and β5i. Compound 1 was predicted to bind to subunits β5 and β5i with energy estimates of −97.03 and −104.72 kJ/mol, respectively. Although energy values are relatively similar for both subunits, there are notable differences in the sites where the compound is predicted to bind (Figure 6).

Compound 1 was predicted to be oriented towards the catalytic site of subunit β5i of the immunoproteasome (Figure 6a), almost fully inserted in the corresponding S1 specificity pocket (Figure 6c). The S1 pocket has been found to be critical in determining selective binding of ligands to subunits β5 and β5i. This pocket also contains an N-terminal Thr1 residue that is essential for the catalytic activity of the subunits [36]. Compound 1 appears to form two hydrogen bonds with residues from the β5i subunit, one between the carbonyl group at C-16 and the Thr1 residue, and another one between the methoxyl group at C-9 and the Gly23 residue (Figure 6a). Conversely, in the subunits of the constitutive proteasome, compound 1 is predicted to bind towards the neighboring β6 subunit, relatively away from the catalytic site of subunit β5 and its S1 pocket (Figure 6b,d). In this case, compound 1 also appears to form a hydrogen bond with Gly23, but involves a carbonyl group at C-20 rather than the methoxyl group at C-9.

**Figure 7:**
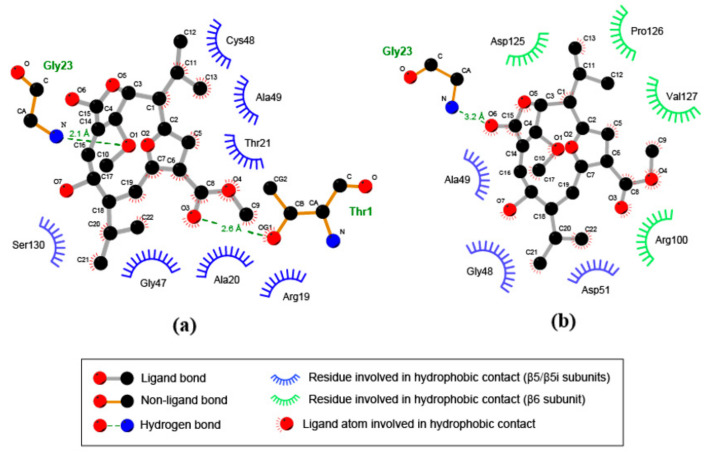
A different pattern of noncovalent interactions is involved in binding of compound 1 to β5 and β5i subunits. Two-dimensional representation of ligand–protein interactions for compound 1 and the β5/β5i–β6 subunits of the murine immunoproteasome (**a**) and the constitutive proteasome (**b**). Atoms are represented by their chemical symbol. For side chains of amino acids, CA, CB and CG indicate the α, β, and γ carbon atoms, respectively.

Further prediction of noncovalent interactions with LigPlot+ revealed that binding of compound 1 to the S1 pocket of the β5i subunit is also facilitated by hydrophobic contacts with several amino acid residues from the pocket (Figure 7a). Among those residues are Ala20 and Ala49, which in turn are involved in the CTL activity of the subunit [36]. Although hydrophobic interactions with residues from the β5 subunit also appear to be relevant for binding of compound 1 to the constitutive proteasome, these involve additional contacts with residues from the neighboring β6 subunit (Figure 7b). These results suggest that compound 1 might modulate the β5i subunit activity due to its specific interaction with amino acids involved in the catalytic activity of the subunit.

We also analyzed the interaction of compound 1 with β1i subunit and with the corresponding β1 subunit of the constitutive proteasome. Compound 1 was predicted to bind to the catalytic site of subunit β1i, while binding to a different but relatively closer site in subunit β1 (Figure S9). Predicted energy values were −102.09 and −106.69 kJ/mol, for the β1 and β1i subunits respectively.

In order to determine the functional groups that could be involved in the interaction of compound 1 to the β5i subunit, we performed docking simulations for the structurally related compound isogorgiacerodiol, which presents a weaker anti-inflammatory activity [16]. This compound differs from compound 1 only in the substitution of the methoxyl group at C-9 by a hydroxyl group (Figure S1–S4). The compound was predicted to bind outside the catalytic site of subunits β5 and β5i (Figure S10). In the immunoproteasome, this compound appears to form a hydrogen bond with its hydroxyl group at C-20 and residue Ala50 of the β5i subunit, which is close to the interface between subunits β5i and β6. Furthermore, orientation of this compound is favored by a pattern of hydrophobic interactions involving residues Asp125 and Pro126 from the β6 subunit. Isogorgiacerodiol does not inhibit the CTL activity in vitro (Figure S10). These results indicate that the methoxyl group at C-9 of compound 1, which is not present in isogorgiacerodiol, may be critical for the orientation that facilitates its binding to the catalytic site of the β5i subunit.

## 4. Discussion

We have previously shown that compound 1 inhibits the inflammatory response induced in macrophages after LPS challenge by a mechanism involving the reduction of IκBα degradation [16]. Since the proteasome is critical for IκBα degradation and activation of NFκB, we suspected that the compound might be interfering with the activity of the proteasome. Here, we found accumulation of polyubiquitinated proteins in murine macrophages stimulated with LPS and treated with compound 1, which correlates with a reduced proteasomal CTL and caspase-like activities. Analyses on purified proteasomes revealed that compound 1 inhibits the CTL activity of immunoproteasome. We also showed that compound 1 reduces the LPS-induced surface expression of MHC class I molecules in vitro and the production of proinflammatory mediators in vivo. Docking simulations have predicted a selective interaction of compound 1 with the β5i subunit. Thus, the anti-inflammatory effect of this compound might be dependent, at least in part, on the modulation of immunoproteasome activity.

It has been previously shown that LPS induces proteasomal activation in immune cells and that a proteasome inhibitor, lactacystin, blocks the expression of multiple genes involved in the response of macrophages to LPS [4]. Hence, we determined the effect of compound 1 on the proteasome activity in LPS-stimulated macrophages. Considering the relevance of chymotrypsin-like activity of proteasomes in the inflammatory response of macrophages [37,38], we evaluated the effect of compound 1 on β1i and β5i immunoproteasome subunits and their counterparts in the constitutive proteasome. We showed herein that compound 1 inhibits the CTL activity of the proteasome, thus resulting in the accumulation of polyubiquitinated proteins. This effect of compound 1 on CTL activity did not occur in the absence of LPS, suggesting that the compound might be modulating the immunoproteasome activity. Compound 1 also inhibited the caspase-like activity of the proteasome; however, this effect did not exclusively occur in the presence of LPS. The inhibition of constitutive proteasomal CTL activity has been previously reported for different plant-derived terpenoids, and this effect is associated with anti-inflammatory and/or anti-cancer properties of these compounds [39,40].

The effect of compound 1 on purified immunoproteasome and constitutive proteasome was further evaluated. While compound 1 inhibited the CTL activity of immunoproteasome at the three concentrations analyzed, the inhibition of the constitutive proteasome only occurred at a higher compound concentration. Previous reports have shown that in RAW 264.7 cells early TNF production induced by LPS is not regulated by the immunoproteasome and that the inhibition of β2 and β5 constitutive proteasome subunits is required for a decrease in the production of this cytokine [5]. However, we have previously demonstrated that compound 1 inhibits the expression and secretion of inflammatory mediators, including TNF, in peritoneal macrophages stimulated with LPS as early as 3 h of stimulus [16]. Differences in the kinetic expression of immunoproteasome subunits induced by LPS in cell lines and primary macrophages might explain these discrepancies.

In immune cells, pro-inflammatory stimuli induce the replacement of the constitutive proteasome by the immunoproteasome, which increases MHC class I antigen processing and regulates inflammatory responses. Stimulation of cells with IFN-γ and TNF leads to the expression of immunoproteasome subunits [26,41]. It has been reported that the expression of LPS-induced immunoproteasome subunits is implicated in the production of certain inflammatory mediators [5]. We have shown that LPS preferentially increased mRNA expression of β1i and β5i compared to β1 and β5 in murine peritoneal macrophages that were not affected by treatment with compound **1**. No expression of mRNA for proteasome subunits was observed in control cells without stimulus. However, β5i protein has been detected in the cytoplasm of nonstimulated peritoneal macrophages [17]. Although we have not found differences in mRNA expression between β1i and β5i in LPS-stimulated macrophages, other authors have reported higher protein levels of β1i than those of β5i after stimulation with IFN-γ [17]. Previous reports of the occurrence of mixed-subunit proteasomes, involving one or two inducible subunits coupled with constitutive ones, may support these findings [12,42]. Further studies are necessary to characterize the proteasome composition influenced by LPS stimulus.

Immunoproteasome has been largely implicated in the processing of MHC class I-restricted peptides [43]. Activation of cytotoxic T lymphocytes depends on the recognition of peptides presented by MHC-I molecules. Immunoproteasome generates peptides for MHC-I presentation more efficiently than the constitutive proteasome, probably by means of the substitution of the caspase-like activity of β1 subunit by the CTL activity of β1i [44]. Proinflammatory stimuli such as IFN-γ, TNF and LPS upregulate the cell surface expression of MHC-I molecules [45,46]. Our results show that LPS induces an increment in the levels of MHC-I in cell surface, an effect that was avoided by compound 1. These results are congruent with the idea that compound 1 might be interfering with immunoproteasome activity, affecting MHC-I expression. Our data are consistent with previous findings in which the inhibition of immunoproteasome subunits by using PR-957 reduces cell surface expression of MHC-I in splenocytes and cytokine production in monocytes [47]. Deficiency of β5i in mice generates a reduction in MHC-I cell surface expression levels compared to wild type mice [2], which was not observed in the absence of β1i [48]. These results point out a role of β5i in MHC class I expression.

In vivo, inhibition of immunoproteasome modulates immune responses and disease progression in several models. Treatment of animals with PR-957 reduces signs of experimental arthritis, which is associated with a reduction in joint expression of proinflammatory mediators [47]. This inhibitor also attenuated the progression of experimental autoimmune encephalomyelitis and prevented the expression of pro-inflammatory mediators in the brain and spinal cord of animals [49]. A mouse colitis model has revealed that the blockage of β5i subunit reduced the pathological sings of the disease and cytokine production in the colon [34]. Immunoproteasome subunits are rapidly induced in lungs after viral infection of mice [50]. Here we have shown that compound 1 inhibited the production of proinflammatory mediators in the lungs and in the bronchoalveolar lavage of mice treated with LPS. This effect could be at least partially dependent on the inhibition of immunoproteasome subunits, since LPS significantly upregulated the expression of β1i and β5i in lungs. Further studies are necessary to demonstrate the interaction of compound 1 with immunoproteasome in vivo.

Molecular docking simulations supported the notion that compound 1 inhibits CTL activity of the immunoproteasome. The compound was predicted to bind to the catalytic site of the β5i subunit, oriented towards its S1 specificity pocket. The compound forms at least two hydrogen bonds with residues from the subunit, one between a methoxyl group and residue Gly23, and another between a carbonyl group and the N-terminal Thr1 residue. Thr1 is actively involved in the catalytic mechanism of the subunit [51,52] and was recently shown to participate in the activation of proteolytic activity during biogenesis of the proteasome [53]. Furthermore, several well-studied proteasome inhibitors, such as PR-957, interact with this residue [36].

Conversely, in the subunits of the constitutive proteasome, compound 1 was predicted to bind towards a small cavity of the neighboring β6 subunit, leaving the S1 pocket empty. The compound also appears to form a hydrogen bond with residue Gly23, but involves a carbonyl group at C-20 rather than a methoxyl group. Taken together with the experimental evidence, these predictions suggest that compound 1 might selectively inhibit the CTL activity of the immunoproteasome, by binding to the β5i subunit. Docking of the structurally-related compound isogorgiacerodiol, which presents a substitution of the methoxyl by a hydroxyl group at C-9, was predicted to bind outside the catalytic site of the β5i subunit, oriented towards the neighboring β6 subunit. These results point out the methoxyl group of compound 1 as critical for the orientation that facilitates its binding to the catalytic site of the β5i subunit. 

Since it has been demonstrated that the β1i subunit of the immunoproteasome has CTL activity, we also evaluated the interaction of compound 1 with this subunit. The compound was also predicted to bind to the catalytic site of the β1i subunit, with binding energy estimates similar to those for the β5i subunit. Results suggest that the effect of the compound on the CTL activity of the immunoproteasome could be the consequence of binding to two subunits with similar activity. This finding is consistent with reported evidence that inhibition of multiple proteolytic sites is needed for a marked reduction of proteasome-mediated proteolysis [54].

Different contributions to inflammatory responses and other functions related to the immune system have been attributed to different proteasome subunits. Deficiency in the β1i subunit induced a reduction of cytokine production by murine B cells stimulated with LPS, through a mechanism partially dependent on NFκB inhibition [12]. Altered NFκB activity in nonobese diabetic mice has been attributed to a defect in proteasome function due to a lack of β1i subunit expression [10]. Selective inhibition of the β5i subunit leads to partial reduction of TNF and IL-6 production induced by LPS in vitro, which is completely abrogated if β1i and β2i are also inhibited [47]. The in vitro and in vivo identification of heterogeneous proteasome populations that differ in their enzymatic features [55] may explain the role attributed to individual proteasome subunits in pathological conditions.

Proteasome inhibitors are promising candidates for the treatment of inflammatory diseases and cancer. Currently, there are three proteasome inhibitors approved by the United States Food and Drug Administration (FDA) for clinical use in humans, namely, bortezomib, carfilzomib and ixazomib. The immunoproteasome is overexpressed in malignant cells and in cells involved in autoimmune disorders [20,56]. Selective inhibitors of the immunoproteasome have the advantage of being effective as a treatment for such conditions, while preventing the onset of undesirable side effects associated with the inhibition of the constitutive proteasome [40]. Here we propose a natural compound as a potential specific inhibitor of the CTL activity of the immunoproteasome, opening a path for further studies to characterize this compound as a new agent for the treatment of inflammatory and autoimmune diseases.

## 5. Conclusions

We have shown here a diterpenoid as a potential inhibitor of CTL activity of the proteasome. This compound inhibited the expression of MHC class I molecules in vitro and reduced the levels of pro-inflammatory cytokines in response to LPS in vivo. Molecular docking has predicted that the compound selectively interacts with the S1 pocket of the β5i subunit of the immunoproteasome, a common feature among immunoproteasome inhibitors. The immunoproteasome has been implicated in the induction and maintenance of inflammation and a more efficient generation of peptides for MHC-I presentation. The immunoproteasome has been proposed as a potential therapeutic target, since it is associated with several pathological conditions including cancer and inflammation. This work suggests that this compound might be a selective inhibitor of the immunoproteasome, hence pointing it out as a candidate in the search for new drugs for the treatment of inflammatory disorders and autoimmune diseases.

## Supplementary Materials

The following are available online at https://www.mdpi.com/2218-273X/8/4/109/s1, Figure S1: ^1^H-NMR and ^13^C-NMR spectra and structure of compound **1,** Figure S2: ^1^H-NMR and ^13^C-NMR spectra and structure of isogorgiacerodiol, Figure S3: HRAPCI-MS spectra and structure of compound **1**, Figure S4: HRAPCI-MS spectra and structure of isogorgiacerodiol, Figure S5: Compound **1** inhibits caspase-like activity but not CTL activity in the absence of LPS, Figure S6: LPS induces the expression of immunoproteasome subunits, Figure S7: IC50 sigmoidal curve of the effect of compound 1 on CTL activity of the immunoproteasome, Figure S8: LPS selectively induces the expression of immunoproteasome subunits in vivo, Figure S9: Predicted orientation of compound **1** within the catalytic sites of subunits β1 and β1i of the murine constitutive and immunoproteasome, respectively, Figure S10: Effect of isogorgiacerodiol on proteasome CTL activity.
