# Supplementary material for: A Marine Diterpenoid Modulates the Proteasome Activity in Murine Macrophages Stimulated with LPS
Source: Biomolecules. 2018 Oct 5;8(4):109. doi: 10.3390/biom8040109 (PMC6315684; doi:10.3390/biom8040109)
Supplement: Supplementary file 1 [file biomolecules-08-00109-s001.pdf]

A marine diterpenoid modulates the proteasome activity in murine macrophages stimulated with LPS

Yisett González, Deborah Doens, Héctor Cruz, Ricardo Santamaría, Marcelino Gutiérrez, Alejandro Llanes*, Patricia L. Fernández*

Supplementary Figures

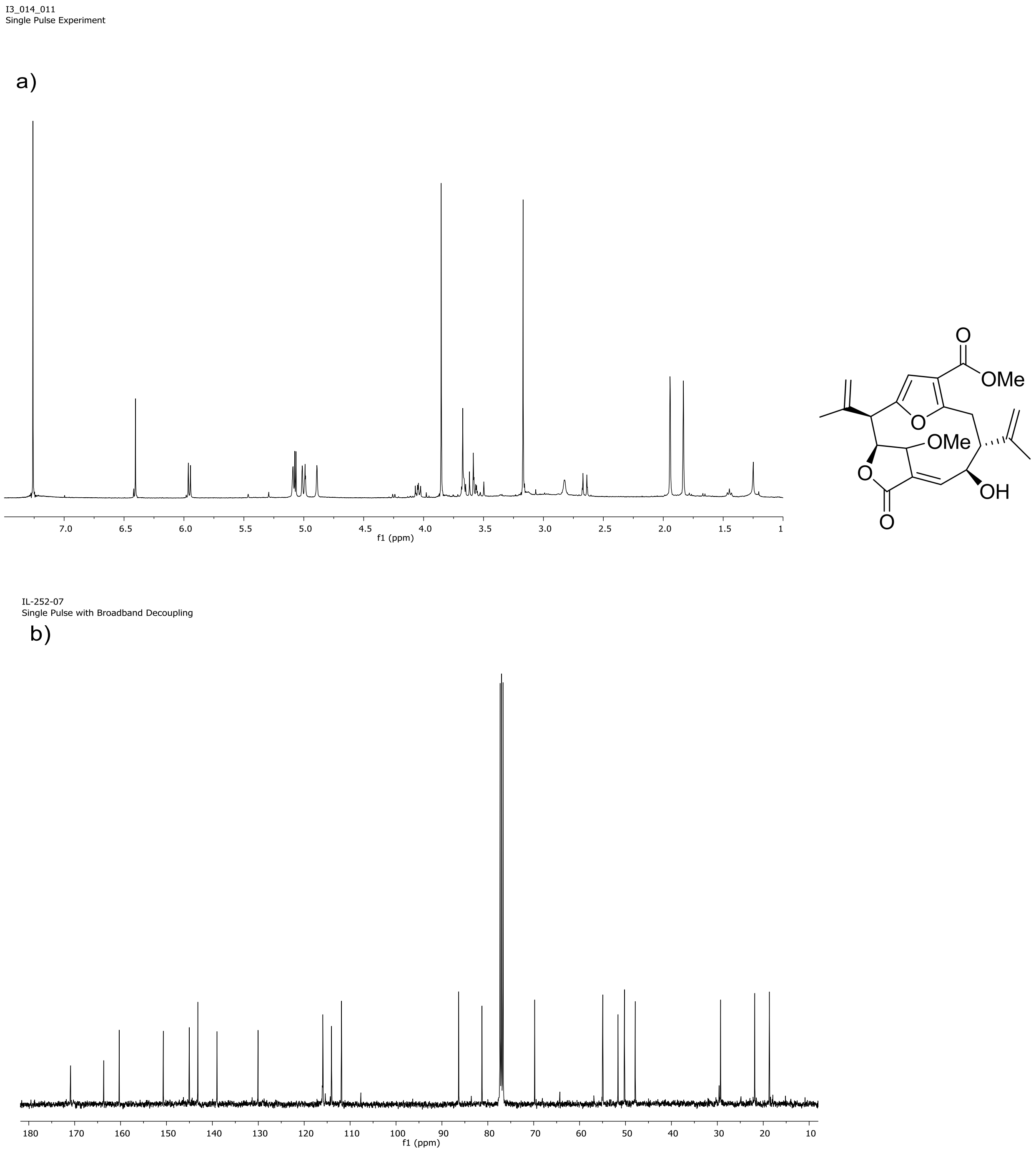

Fig. S1. ^{1}H-NMR (a) and ^{13}C-NMR (b) spectra and structure of compound **1** isolated from the octocoral *Pseudopterogorgia acerosa*. For details on isolation and purification processes see reference 16 from the article.

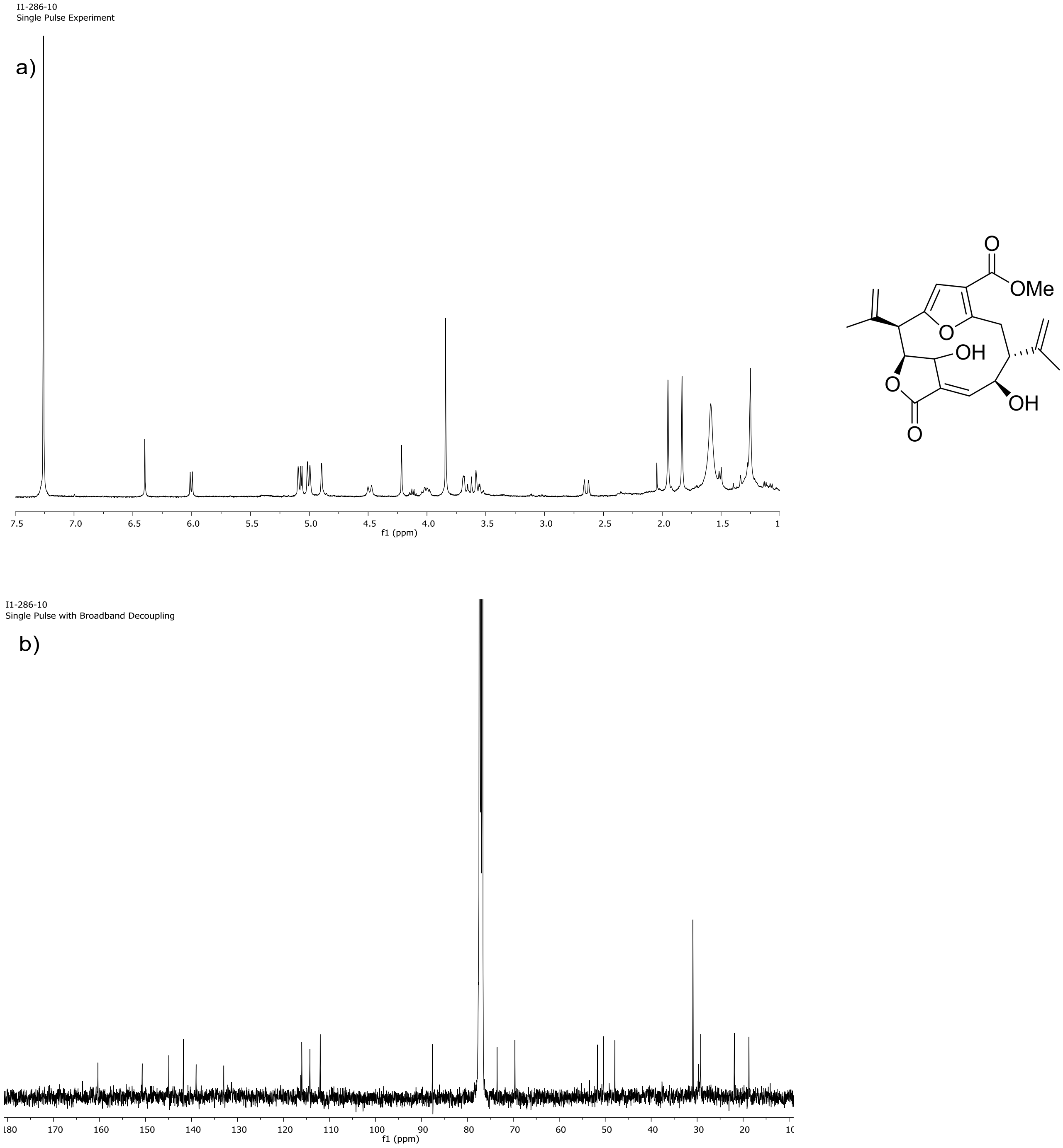

Fig. S2. ^{1}H-NMR (a) and ^{13}C-NMR (b) spectra and structure of isogorgiacerodiol isolated from the octocoral *Pseudopterogorgia acerosa*. For details on isolation and purification processes see reference 16 from the article.

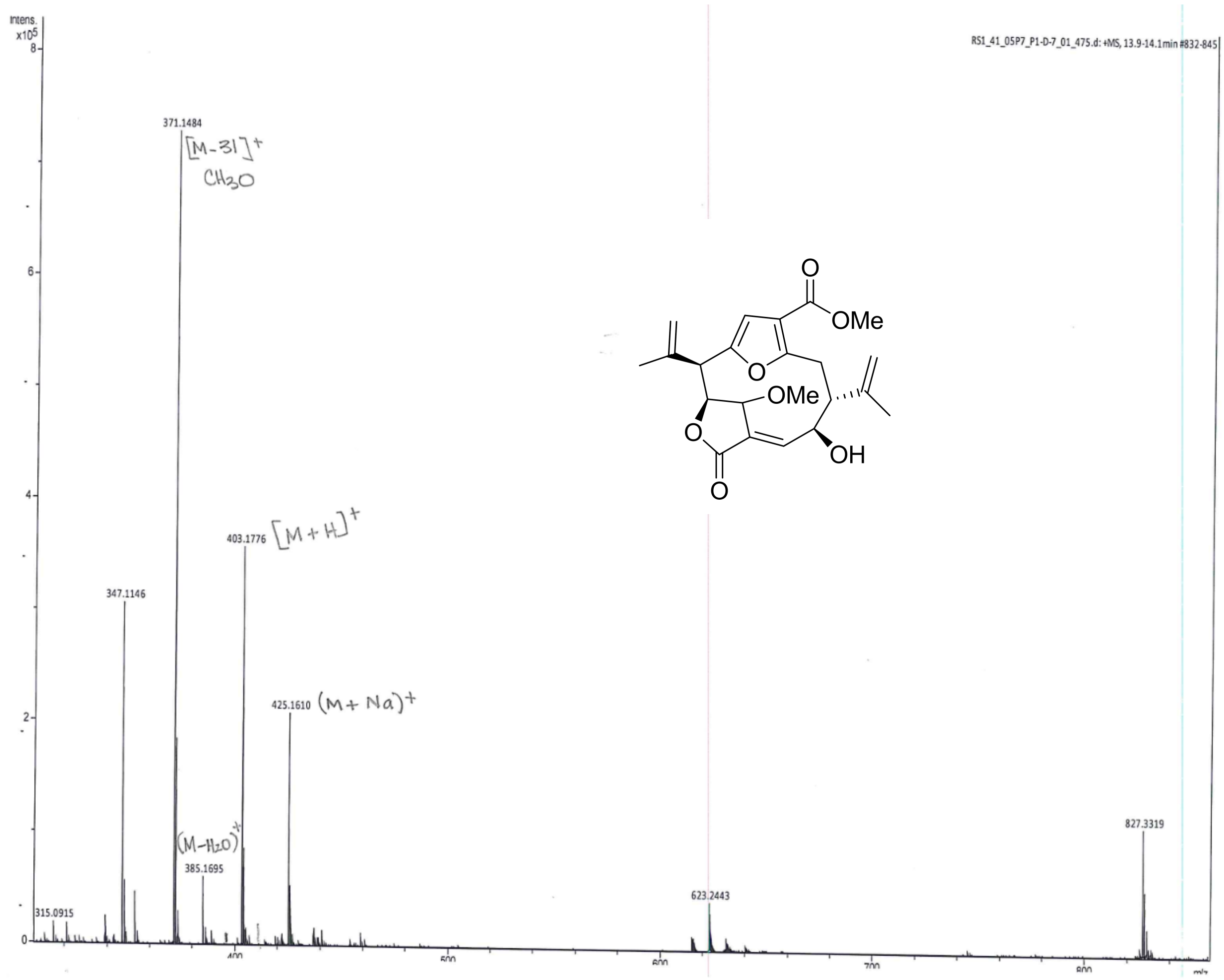

Fig. S3. HRAPCI-MS spectra and structure of compound **1** isolated from the octocoral *Pseudopterogorgia acerosa*.

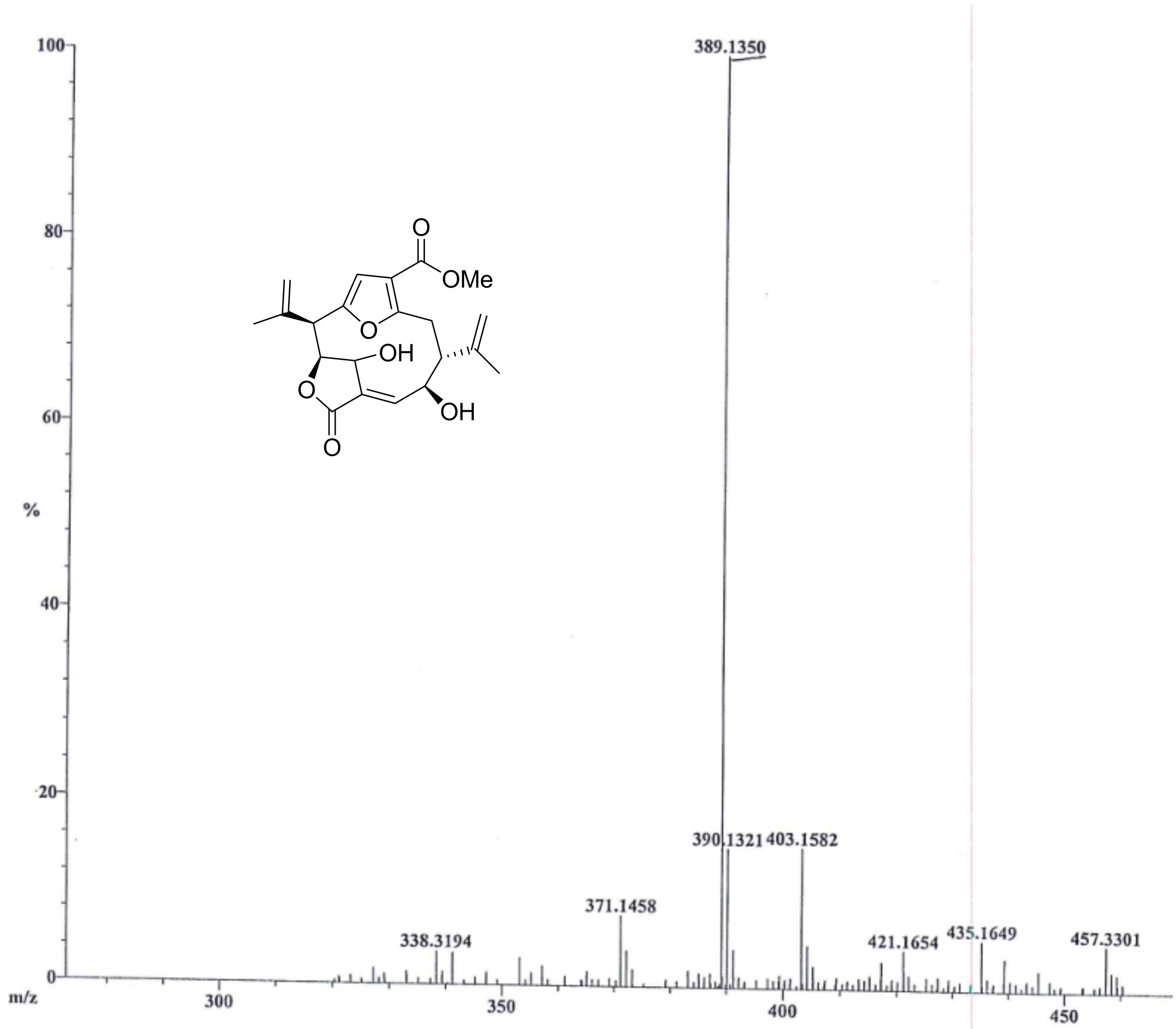

Fig. S4. HRAPCI-MS spectra and structure of isogorgiacerodiol isolated from the octocoral *Pseudopterogorgia acerosa*.

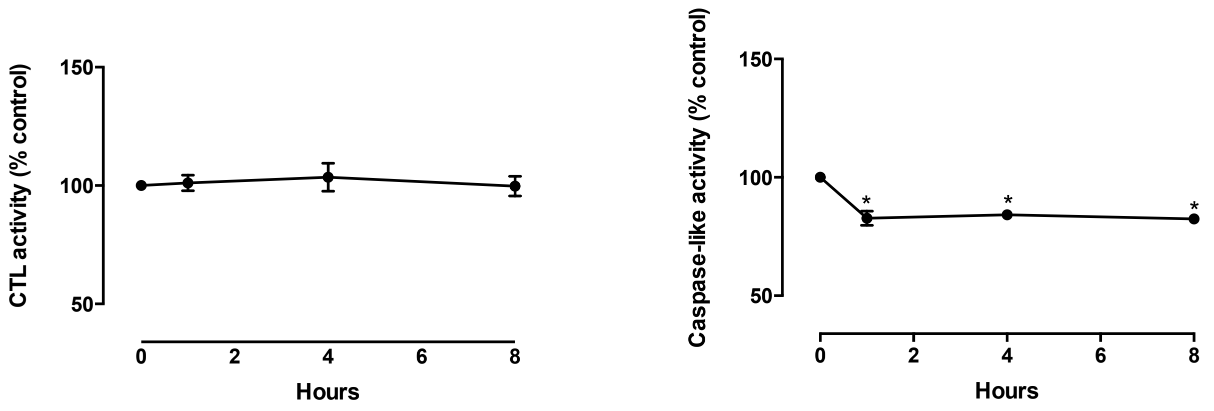

Fig. S5: Compound **1** inhibits caspase-like activity but not CTL activity in the absence of LPS. Peritoneal macrophages were treated with compound 1 (25 μM) for 2, 4 or 8 hours. Hydrolysis of fluorogenic peptides Suc-Leu-Leu-Val-Tyr-AMC (left panel) or Z-Leu-Leu-glu-AMC (right panel) was measured in cell supernatants by detection of free AMC. Results were normalized with DMSO-treated controls. Results represent mean ± s.d. from treatments performed in triplicated and are representative of two different experiments.

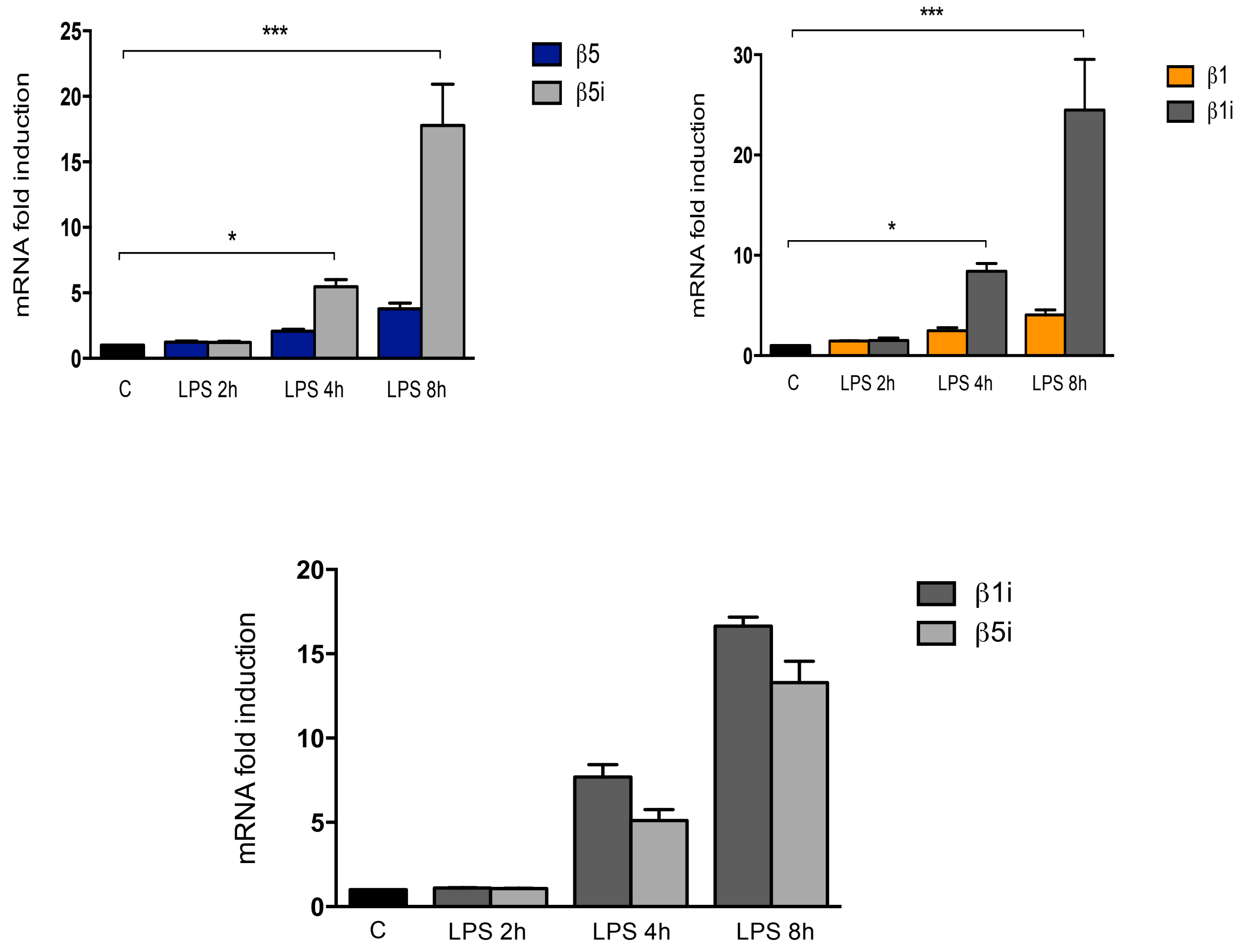

Fig S6: LPS induces the expression of immunoproteasome subunits. Peritoneal macrophages were treated with LPS for 2, 4 or 8 hours and mRNA levels of β1, β1i, β5 and β5i were determined by quantitative PCR. Results were normalized to HPRT expression and are presented as fold induction of mRNA expression relative to control samples. Results represent means ± s.e.m. from two independent experiments performed in duplicates. *, $P < 0.05$; ***, $P < 0.001$.

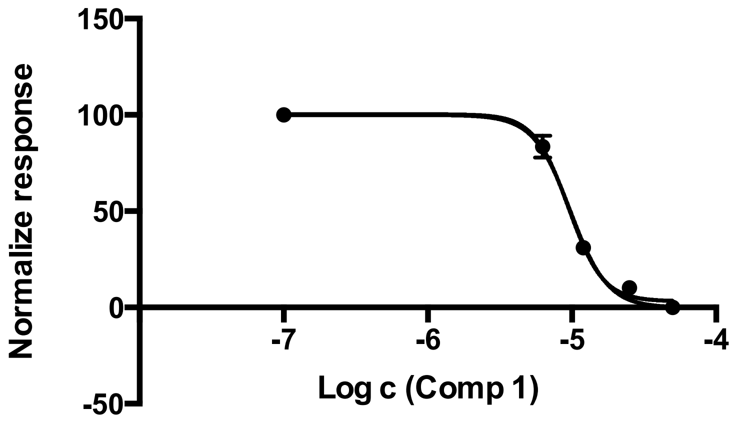

Fig S7: IC_{50} sigmoidal curve of the effect of compound **1** on CTL activity of the immunoproteasome. IC_{50} sigmoidal curve calculated by the statistical software package GraphPad Prism 6. Results represent means ± S.D. from samples assayed in triplicate.

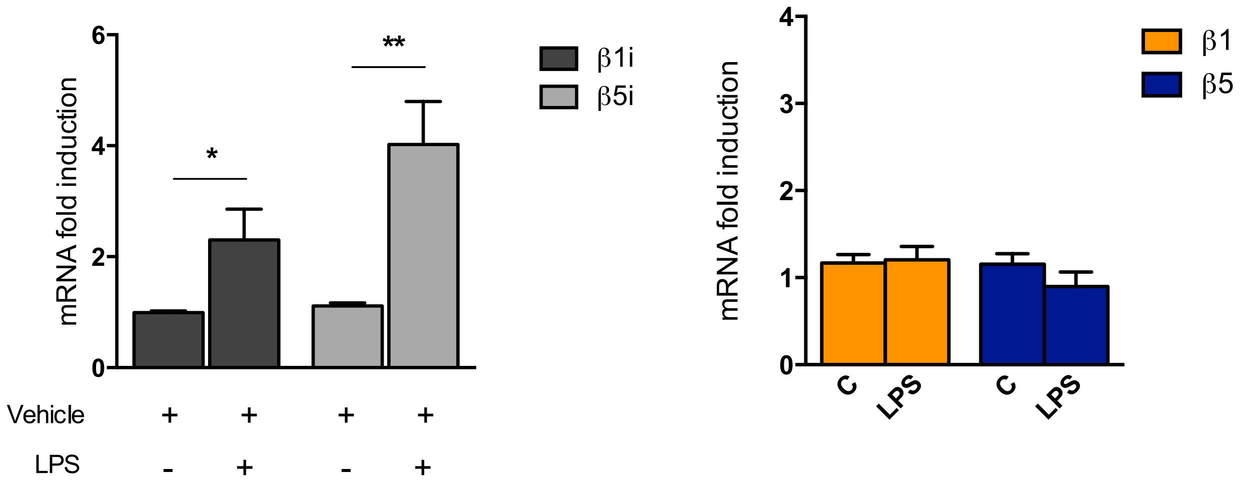

Fig S8. LPS selectively induces the expression of immunoproteasome subunits *in vivo.* C57Bl/6 mice were treated with LPS (0.5 mg/Kg) or vehicle by intranasal inoculation. Twenty-four hours later animals were euthanized and mRNA expression of β1i, β5i ,β1 and β5 were determined in lungs. Results represent mean ± s.e.m from two different experiments. *, P< 0.05; **, P< 0.01.

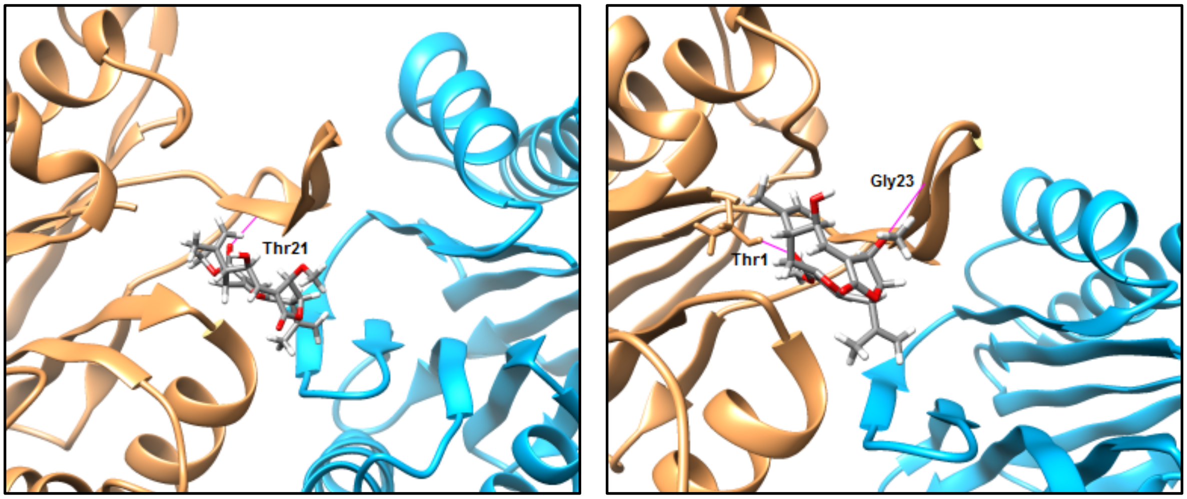

(a) **(b)**

Fig. S9. Predicted orientation of compound **1** within the catalytic sites of subunits β1 **(a)** and β1i **(b)** of the murine constitutive and immunoproteasome, respectively. Neighboring β2 and β2i subunits were included to assess their contribution to interactions within β1 and β1i catalytic sites. β1/β1i subunits are colored orange and β2/β2i subunits are colored light blue. Predicted hydrogen bonds between the ligand and amino acid residues from those subunits are indicated by purple lines.

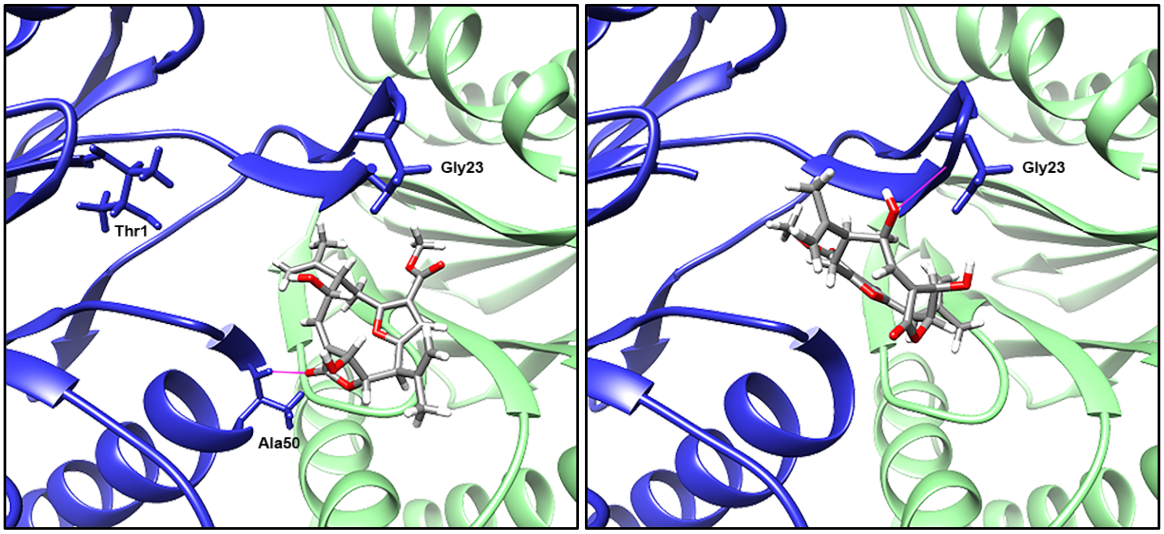

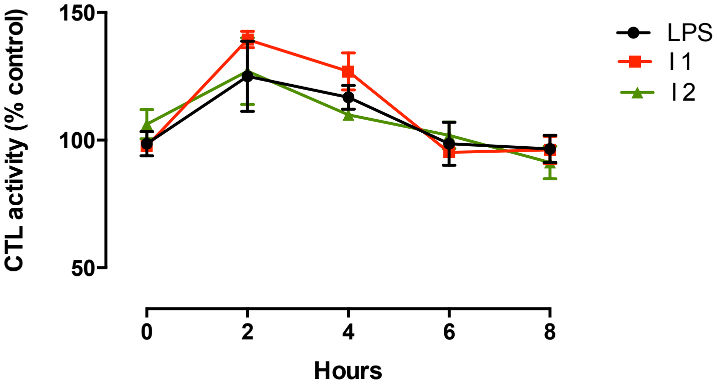

Fig. S10. Effect of isogorgiacerodiol on proteasome CTL activity. Predicted orientation of isogorgiacerodiol within the dimer of β5/β5i and β6 subunits of the murine immunoproteasome (left) and the constitutive form (right). Subunits β5/β5i are colored blue and β6 subunits are colored green. Predicted hydrogen bonds between the ligand and amino acid residues from those subunits are indicated by purple lines. Macrophages were stimulated with LPS (1 µg/mL) in the presence or absence of 25 µM (I 1) or 50 µM (I 2) of Isogorgiacerodiol for 2, 4, 6 or 8 h. Hydrolysis of fluorogenic peptide Suc-Leu-Leu-Val-Tyr-AMC was measured in cell supernatants by detection of free AMC. Results were normalized with DMSO-treated controls. Results represent mean ± s.e.m from two independent experiments.
